# On the Relation of Peliosis to the Other Forms of Idiopathic Purpura

**Published:** 1896-01

**Authors:** Alfred E. Diehl

**Affiliations:** Buffalo, N. Y.


### ON THE RELATION OF PELIOSIS TO THE OTHER FORMS OF IDIOPATHIC PURPURA.

BY ALFRED E. DIEHL, A. B., M. D., Buffalo, N. Y.

EVER since the classification of diseases of the skin by Hebra, there has been more or less discussion as to the position which peliosis or purpura rheumatica should occupy. While nearly all writers agree that purpura simplex and purpura hemorrhagica are one and the same affection, differing only in the degree of severity, yet there is a difference of opinion as to what relation peliosis holds in connection with these two. Some writers hold that it is a separate disease; others, that it is one of the exudative erythemata and some hold that it is the same disease as purpura simplex with the addition of the joint symptoms.

The following case, I think, goes to prove the last theory, *i. e.*, that purpura simplex, peliosis and purpura hemorrhagica are one and the same affection, each presenting the same local manifestations and differing only in their individual severity.

The affection in this case began as a simplex purpura and continued as such for some length of time and apparently recovering, but a sudden change of atmospheric conditions again brought out the manifestations of the disease with increased severity, thus showing a distinct rheumatic tendency.

The case is as follows:

Miss M., aged 28 years, family history was negative, never had rheumatism, nor was there a rheumatic diathesis. On January, 1895, she first noticed a few pin's head sized spots on the inner surface of both thighs. These spots covered an area equal to about the size of a palm of the hand. At that time she had no fever, no symptoms of debility, no appreciable anemia, appetite was good, in fact, as she said, she felt perfectly well. Further examination showed no organic disease whatever. During the course of a few days these purpuric spots appeared with increasing frequency and on the evening of January 13th her legs became swollen and extremely painful, necessitating her being carried up stairs. Inspection at the time showed her legs and thighs to be literally covered with purpuric spots, ranging in size from a pin's head to that of a ten cent piece. There were also a few petechiæ on the arms, but none whatever on the trunk. The only symptom the patient complained of was the pain and swelling of the legs. Under treatment and absolute rest in bed, these symptoms gradually disappeared and at the end of a week there was not a sign of any hemorrhage whatever; consequently she was allowed to sit up. This, however, at once brought on another severe eruption. She was then ordered in bed again under the same restrictions as at first and kept there until April 10th. During this time several purpuric eruptions occurred, but not any of a severe character. She was then allowed to sit up in bed and for some days no hemorrhages occurred. It was then thought possible that she could go into the country, without danger to herself, in search of rest and fresh air. From that time until May 13th she remained entirely free from all hemorrhages, but on that day there was a severe frost and while out driving a severe eruption again came out on the legs accompanied with great pain and swelling, necessitating her being lifted from the carriage. She soon recovered from this attack and from then until the present time she has had no further attacks, with the exception of a few occasional petechiæ.

362 Pearl Street.

